# A Simulation-Based Assessment of Strategies to Control *Clostridium Difficile* Transmission and Infection

**DOI:** 10.1371/journal.pone.0080671

**Published:** 2013-11-21

**Authors:** Michael A. Rubin, Makoto Jones, Molly Leecaster, Karim Khader, Willy Ray, Angela Huttner, Benedikt Huttner, Damon Toth, Theodore Sablay, Robert J. Borotkanics, Dale N. Gerding, Matthew H. Samore

**Affiliations:** 1 Department of Internal Medicine, George E. Wahlen Department of Veterans Affairs Medical Center, Salt Lake City, Utah, United States of America; 2 Department of Internal Medicine, University of Utah School of Medicine, Salt Lake City, Utah, United States of America; 3 Infection Control Program, Geneva University Hospitals, Geneva, Switzerland; 4 Department of Environmental Health Sciences, Johns Hopkins Bloomberg School of Public Health, Baltimore, Maryland, United States of America; 5 Department of Internal Medicine, Edward Hines Jr. Department of Veterans Affairs Hospital, Hines, Illinois, United States of America; University of Cambridge, United Kingdom

## Abstract

**Background:**

*Clostridium difficile* is one of the most common and important nosocomial pathogens, causing severe gastrointestinal disease in hospitalized patients. Although "bundled" interventions have been proposed and promoted, optimal control strategies remain unknown.

**Methods:**

We designed an agent-based computer simulation of nosocomial *C. difficile* transmission and infection, which included components such as: patients and health care workers, and their interactions; room contamination via *C. difficile* shedding; *C. difficile* hand carriage and removal via hand hygiene; patient acquisition of *C. difficile* via contact with contaminated rooms or health care workers; and patient antimicrobial use. We then introduced six interventions, alone and "bundled" together: aggressive *C. difficile* testing; empiric isolation and treatment of symptomatic patients; improved adherence to hand hygiene and contact precautions; improved use of soap and water for hand hygiene; and improved environmental cleaning. All interventions were tested using values representing base-case, typical intervention, and optimal intervention scenarios.

**Findings:**

In the base-case scenario, *C. difficile* infection rates ranged from 8–21 cases/10,000 patient-days, with a case detection fraction between 32%–50%. Implementing the "bundle" at typical intervention levels had a large impact on *C. difficile* acquisition and infection rates, although intensifying the intervention to optimal levels had much less additional impact. Most of the impact came from improved hand hygiene and empiric isolation and treatment of suspected *C. difficile* cases.

**Conclusion:**

A "bundled" intervention is likely to reduce nosocomial *C. difficile* infection rates, even under typical implementation conditions. Real-world implementation of the "bundle" should focus on those components of the intervention that are likely to produce the greatest impact on *C. difficile* infection rates, such as hand hygiene and empiric isolation and treatment of suspected cases.

## Introduction

*Clostridium difficile* infection (CDI) is one of the most common and important healthcare-associated infections (HAI) among patients hospitalized in the United States and Europe,[1]–[3] producing a spectrum of clinical diseases ranging from asymptomatic colonization to life-threatening toxic megacolon.[3], [4] *C. difficile* is acquired exogenously, similar to other enteric pathogens, with the principal reservoirs of infection thought to be colonized or infected individuals and contaminated environments such as hospitals and chronic care facilities.[2], [4] The high cost of care associated with CDI[4]–[6] underscores the urgent need for improved hospital infection control and prevention practice. This is particularly true given the recent emergence of an epidemic strain of *C. difficile* (BI/NAP1/027) that is associated with higher mortality and morbidity, especially in older patients.[6]

Despite progress in defining the mechanisms of *C. difficile* transmission and interventions to control its spread in endemic settings,[7]–[9] optimal control strategies for *C. difficile* remain unclear.[10] Many of these studies have limited generalizablility due to differences in study populations, contact networks, antimicrobial exposure, and predominant strain of *C. difficile*. In fact, recently published clinical practice guidelines for CDI from the Society for Healthcare Epidemiology of America and the Infectious Diseases Society of America include only two recommendations based on level I evidence.[11] Despite an urgent need to improve the evidence base, prospective, head-to-head comparisons of infection control practices are prohibitively difficult and expensive to perform. The cost and challenge of effectively controlling for complex interactions among important environmental and intervention factors have impeded definitive studies.

Here, we sought to address current challenges in the control of this pathogen through computer simulation modeling. Simulations are useful for studying complex systems involving multiple dynamic interactions among and between individuals and their environments over time.[12] Models that synthesize current best evidence allow us to replicate known behaviors and occasionally discover non-intuitive relationships. Simulations also provide inexpensive laboratories where decision makers can conduct experiments using scenarios that might be infeasible, unethical, or too expensive to test in the real world. Simulation models have been applied successfully to a number of disease paradigms such as influenza[13], [14] but few thus far have attempted to incorporate the unique epidemiology of *C. difficile*,[15]–[18] particularly with respect to the key role of antibiotics in susceptibility and transmission and the role of environmental shedding and acquisition. We developed an agent-based simulation model of a hospital setting to investigate the degree to which current and novel CDI control strategies, alone and in combination, can decrease *C. difficile* transmission and infection. We hypothesized that "bundled" interventions would decrease nosocomial CDI, but that the individual intervention components would vary in their level of influence and effectiveness.

## Methods

### Approach

We used a type of computer simulation modeling called agent-based modeling (ABM), a technique in which a system is modeled as collections of autonomous entities called agents.[12] Each agent is assigned internal states and behaviors; interactions between agents can then produce emergent, system-level dynamics. Our purpose was to simulate a typical hospital environment, composed of intensive care and non-intensive care units and staffed by physicians and nurses. Within this virtual hospital, we introduced the capacity for *C. difficile* to be spread between patients via contaminated environmental surfaces and health care worker (HCW) hands. Testing and treatment of symptomatic patients was also represented. The model thus served as the framework for the evaluation of infection control interventions aimed at reducing the risk of *C. difficile* transmission.

### Model components and processes

The agent-based model was constructed using Anylogic 6 (XJ Technologies, St. Petersburg, Russia), a Java-based modeling application. The model was engineered to be modular in order to enhance its ability to represent diverse facilities and intervention scenarios. Mathematical formulations associated with each sub-model are presented in detail in Appendix S1.

**Figure 1:**
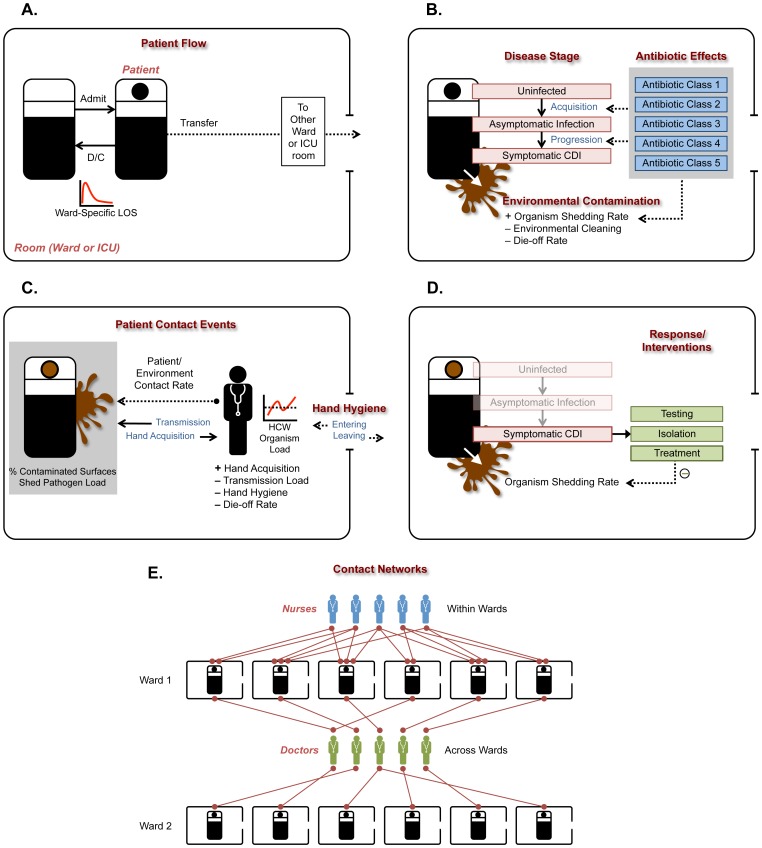
Representations of key agents and sub-models in the simulation, with their interrelationships. Abbreviations: ICU, intensive care unit; LOS, length of stay; HCW, health care worker; CDI, Clostridium difficile infection.

The *patient flow* sub-model (Figure 1A) governed processes of patient admission, transfer, and discharge. The virtual hospital, representing a medium-sized facility, contained nine 30-bed acute care wards and two 15-bed intensive care units. All rooms were single occupancy. Parameters regulating length of stay and time to inter-ward transfers were derived from Veterans Affairs data (see Appendix S1).

The *patient states* sub-model (Figure 1B) included representations of infection status, symptoms, and antimicrobial use. "Susceptible" (non-colonized) patients transitioned to "asymptomatic *C. difficile* infection" as a consequence of acquisition, and "asymptomatic" patients transitioned to "diarrhea due to *C. difficile* infection" as a consequence of progression. Fifty percent of imported *C. difficile* colonization was with a toxigenic strain;[19], [20] both toxigenic and non-toxigenic *C. difficile* caused asymptomatic carriage but only toxigenic *C. difficile* caused diarrhea. Antimicrobial drugs were grouped into five distinct classes to represent differential effects on risk of *C. difficile* acquisition, progression to symptomatic CDI, and organism shedding.

The *contact event* sub-model (Figure 1C) governed processes of contamination of environmental surfaces within the room, transfer of organisms to HCW hands, and acquisition of organisms by susceptible patients. The level of environmental contamination was a function of organism shedding while the room was occupied by an infected patient, and was represented both by pathogen load and the fraction of contaminated surfaces in the room. Symptomatic patients shed organisms at higher rates than asymptomatic patients; outcomes were not sensitive to the ratio of these rates across a wide range of values. Terminal (deep) cleaning reduced a greater fraction of organisms and contaminated surfaces than routine cleaning. HCWs had the potential to acquire *C. difficile* on their hands when they visited rooms occupied by infected patients or rooms occupied by uninfected patients but still contaminated from previous occupants. Hand contamination was a function of the load of organisms in the environment and the infection control practices of the HCW, namely, their hand hygiene behavior and use of barrier precautions (gloves). Susceptible patients acquired *C. difficile* either because of occupancy of a contaminated room or contact with a contaminated HCW.

The *response and intervention* sub-model (Figure 1D) governed policies for managing infected patients and preventing transmission. CDI cases were identified through diagnostic testing. The accuracy of the test was set by sensitivity and specificity parameters; values for a typical qualitative *C. difficile* toxin enzyme immunoassay (EIA) were selected.[21] Patients with CDI were placed on contact isolation. It was assumed that adherence to barrier precautions led to reduced hand contamination when HCWs visited an infected patient or contaminated room. Treatment of patients with CDI led to resolution of symptoms and decreased shedding into the environment.

The *contact network* sub-model (Figure 1E) represented the connections between patients and the two types of HCWs, nurses and physicians. Each patient was assigned a random number of nurses within their ward using a shifted Poisson distribution (having a minimum of 2 and mean of 4) as well as two physicians; nurses only contacted patients within their own ward, while physicians contacted a larger panel of patients across multiple wards. Assignments changed twice daily. Nurses and physicians differed in their frequency of contacts with patients.

### Model parameters

We followed a systematic process to parameterize and calibrate the model. First, an extensive literature search was performed. When available, national and local data were used to generate estimates. When no data were available to inform parameterization, we utilized a Delphi approach[22] on a convenience sample of experts, modified to include face-to-face panel discussions. Candidate point estimates and ranges for these parameters were first established; we then convened our expert panel to solicit their parameter value estimates. Following a period of facilitated debate, the experts were encouraged to revise their estimates based on the evidence presented. Median values of the revised estimates were used to inform the model. A summary of the key parameters used in the model is shown in Table 1.

**Table 1:** Key parameters used in the model, including the parameters governing the six bundle intervention components, the values used for the BASE, INT, and OPT scenarios, and the values used for the epidemiologic conditions.

Parameters for sub-models									
	*Sub-model*							BASE	REF
	Patient states								
		Fraction of new admissions already colonized with *C. difficile*						0.075	[28],EX
		Fraction of new admissions who have symptomatic *C. difficile* at entry						0.0075	[29],EX
		Fraction of new admissions who are already on antibiotics						0.24	DA
		Fraction of imported *C. difficile* strains that are toxigenic						0.50	[19], [20],EX
		Average time from acquisition to development of symptoms (days)						4	[30], [31]
		Average time from development of symptoms to recognition (days)						1.5	[32]–[34]
		Fraction of patients who will start antibiotics per hospital day						0.10–0.12	DA
		Fraction of patients who will stop antibiotics per hospital day						0.33	DA
	Contact events								
		Average number of contacts a patient will have with a doctor per day						4	[35]–[37],DA
		Average number of contacts a patient will have with a nurse per day						20	[35]–[37],DA
		Percent decrease in spores on hands following HH with ABHR						20	[38], [39]
		Percent decrease in spores on hands following HH with soap and water						90	[38], [39]
	Response/Interventions								
		*C. difficile* test sensitivity						0.70	[21], [40]
		*C. difficile* test specificity						0.97	[21]
		Average laboratory turnaround time, EIA test (hours)						2	[40], [41]
		Average time from test order to initiation of contact isolation (days)						1.75	[42], [43],EX
	Contact networks								
		Number of doctors connected to each patient						2	DA
		Number of nurses connected to each patient per nursing shift, mean						4	DA
		Minimum number of nurses connected to each patient per shift						2	DA
**Bundled Intervention**									
	***Intervention Component***					**BASE**	**INT**	**OPT**	**REF**
	Hand hygiene adherence								
		*(% adherence in non-isolation rooms/isolation rooms)*							
		Nurses, before patient contact				30/50	60/70	80/90	[44]–[47]
		Nurses, after patient contact				50/70	70/80	90/90	[44]–[47]
		Physicians, before patient contact				20/30	50/60	70/70	[44]–[47]
		Physicians, after patient contact				40/50	70/80	80/80	[44]–[47]
	Use of soap & water for hand hygiene for CDI patients								
		*(% use of soap & water when HH performed)*				60	80	90	DA,EX
	Use of contact precautions in isolation rooms								
		*(% adherence)*				60	75	90	[48]
	Environmental decontamination								
		*(% organism reduction)*							
		Routine Daily cleaning (*e.g.*, detergent-based)				27.5	30	35	[49]–[53],EX
		Routine Terminal cleaning (*e.g.*, detergent-based)				35	40	42.5	[49]–[53],EX
		Deep Terminal cleaning (*e.g.*, chlorine-based)				70	80	90	[49]–[53],EX
	Aggressive/early testing for CDI								
		*(mean days from symptoms to test order)*				1.5	1	0.5	[32]–[34]
	Empiric isolation and treatment of suspected CDI					No	Yes	Yes	--
**Epidemiologic Conditions**									
	***Condition***					**LOW**	**BASE**	**HIGH**	**REF**
	*C. difficile* importation prevalence *(%)*					2	7.5	15	[28],EX
	*C. difficile* transmission rate					*see Methods for details			DA,EX

Abbreviations: BASE, Base-case values; INT, Typical intervention values; OPT, Optimal intervention values; LOW, low-level values; HIGH, high-level values; HH, hand hygiene; ABHR, Alcohol-based hand rub; EIA, Enzyme immunoassay; abx, antibiotics; REF, reference(s); DA, analysis of local or national VA data; EX, subject matter expert opinion.

### Experimental design

Our goal was to explore the impact of the following six infection control interventions and policies for reducing *C. difficile* transmission and infection:

1. Improved adherence with hand hygiene;
2. Improved use of soap and water for hand hygiene for contacts with CDI patients;
3. Improved adherence with contact precautions for contacts with CDI patients;
4. Improved environmental decontamination;
5. Aggressive/early testing for *C. difficile*;
6. Empiric isolation and treatment of suspected cases of CDI.

These six interventions were studied individually and in combination (as a *C. difficile* “bundle”), across varying levels of implementation. We initially set all intervention parameters at *base-case* (BASE) values; *i.e.*, values that reflect current realities in a typical hospital not employing interventions specifically targeting *C. difficile*. In addition to BASE values, we defined two additional experimental scenarios: (1) *intervention* values (INT), which represent an improvement over BASE values, reflecting the reasonably expected effect from typical adherence to a hospital-wide effort focusing on that particular strategy; and (2) *maximum feasible or optimal* values (OPT), which represent the maximum effects that can be reasonably expected from strong adherence to an intensive and aggressive campaign to reduce *C. difficile* transmission. The parameters governing the six intervention strategies, and the values used for the three experimental scenarios (BASE, INT, and OPT), are shown in Table 1.

For all simulation runs, the primary outcome of interest was incident, nosocomial symptomatic CDI per 10,000 patient-days (PD), in terms of both the actual (true) infection rate and the reported (real-world) rate as observed from positive results of *C. difficile* testing. Secondary outcomes included the rate of *C. difficile* acquisitions (per 10,000 PD); the number of days symptomatic with CDI prior to treatment initiation (per 10,000 PD); the fraction of actual CDI cases detected; the daily *C. difficile* colonization prevalence; and the number of *C. difficile* tests ordered (per 10,000 PD).

To assess the three experimental scenarios (BASE, INT, and OPT), we ran a total of 2000 iterations for each. To assess the differential effect of each bundle component, each of the 486 combinations of component values (3 levels for each component except empiric isolation and treatment, which had 2 levels) were run 39 times.

Experimental scenarios were explored across three sets of epidemiologic conditions representing different levels of *C. difficile* importation and transmissibility: *base-case*, or typical, levels for each (BASE), *high* levels for each (HIGH), and *low* levels for each (LOW). The purpose of this comparison was to assess whether the impact of various infection control measures varied under different epidemiologic conditions. The parameter values used to represent these epidemiologic conditions for importation levels are shown in Table 1. To vary *C. difficile* transmissibility, we utilized a transmissibility parameter as a component of the expression that governs the probability of transmission during a HCW-patient contact event (see Appendix S1). The value of the parameter was tuned to produce an appropriate calibrated output for *C. difficile* aquisition and infection rates for the base-case scenario, which was then adjusted for the LOW and HIGH scenarios. We included 500 runs for each condition for each of the three scenarios.

### Data and statistical analysis

The differences in outcomes among the three experimental scenarios (BASE, INT, and OPT) were assessed graphically using boxplots of rates and also comparing estimated rates of actual and reported CDI. The differences were also assessed graphically for epidemiologic conditions with low values of probability of importation and transmission parameter (LOW) and for high values of both (HIGH).

The impact of independently increasing each single bundle component's value from BASE to INT level (or INT to OPT level) on outcome measures was assessed. A step 1 scenario was defined by setting the component with the greatest single effect at INT level and the other components at BASE level. This step 1 scenario was used as the new baseline from which to assess the impact of independently increasing each single bundle component's value from its current level to the next higher level. This step-wise process was continued until there was no discernible effect of increasing the level of any single component. The assessment was based on estimated CDI rates at each step and also boxplots of actual and reported CDI rates.

### Ethics statement

This study was approved by the Institutional Review Board at the University of Utah as well as by the Research and Development Committee at VA Salt Lake City Health Care System.

## Results

### Outputs of base-case scenario

Base-case parameter values were used in 1,000 simulation runs of one-year periods each. Rates of CDI varied from 8 to 21 per 10,000 PD. The interquartile range was 13 to 16. Reported CDI rates ranged from 5 to 13 per 10,000 PD, with a detection fraction that varied between 32% and 50%. The estimated daily prevalence of toxigenic strains ranged from 8% to 12% and of non-toxigenic strains from 5% to 7%. The difference in colonization prevalence between toxigenic and non-toxigenic strains was due to the higher transmissibility (and shedding) of symptomatic patients.

The rate of acquisition of *C. difficile* ranged from 115 to 202 events per 10,000 PD. The interquartile range was 144 to 160. Approximately 40%of these acquisitions originated from the rooms of symptomatic CDI patients (6% of carriers) and 60% originated from the rooms of asymptomatic carriers (94% of carriers).

The percent of *C. difficile* carriers who progressed to symptomatic CDI varied from 3% to 13% for those who acquired *C. difficile* in the hospital and was approximately 2% to 6% for those who imported *C. difficile*. The lower rate of progression for patients who imported was because (a) half of importation strains were non-toxigenic[19], [20] and (b) patients who imported toxigenic strains were less likely to progress to symptoms than patients who acquired toxigenic strains[20] as specified in the model.

### Impact of bundle

**Figure 2:**
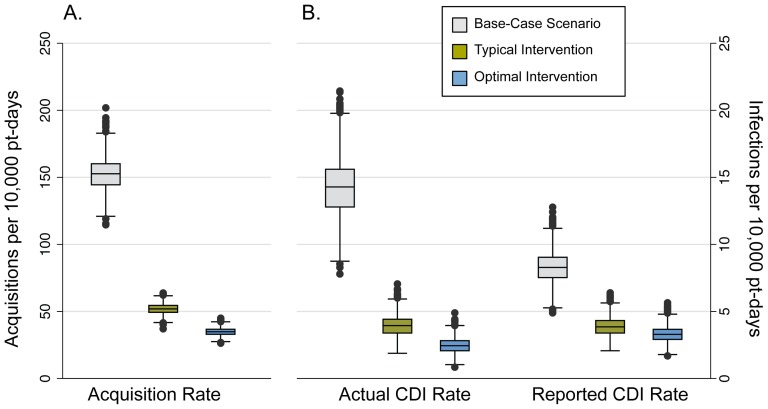
Results of the simulation for the three bundled intervention scenarios. Box plots of (A) *C. difficile* acquisition rate, and (B) actual CDI rate (left) and reported CDI rate (right) for the three bundled intervention scenarios. Grey boxes represent the Base-Case scenario (BASE), green boxes represent the Typical Intervention scenario (INT), and the blue boxes represent the Optimal Intervention scenario (OPT). Rates shown are counts per 10,000 patient-days. The horizontal line within each box represents the median. The top and bottom of each box represent the 25th and 75th percentiles, respectively, and the bars represent the highest and lowest values within 1.5 times the interquartile range. The circles denote outliers.

Implementing the bundle at INT levels had a large impact on *C. difficile* acquisition rates (Figure 2A), as well as on both actual and reported CDI rates (Figure 2B). The intensification of intervention levels to OPT reduced the acquisition and actual CDI rates, to a small extent, but had little to no effect on the reported CDI rate.

**Figure 3:**
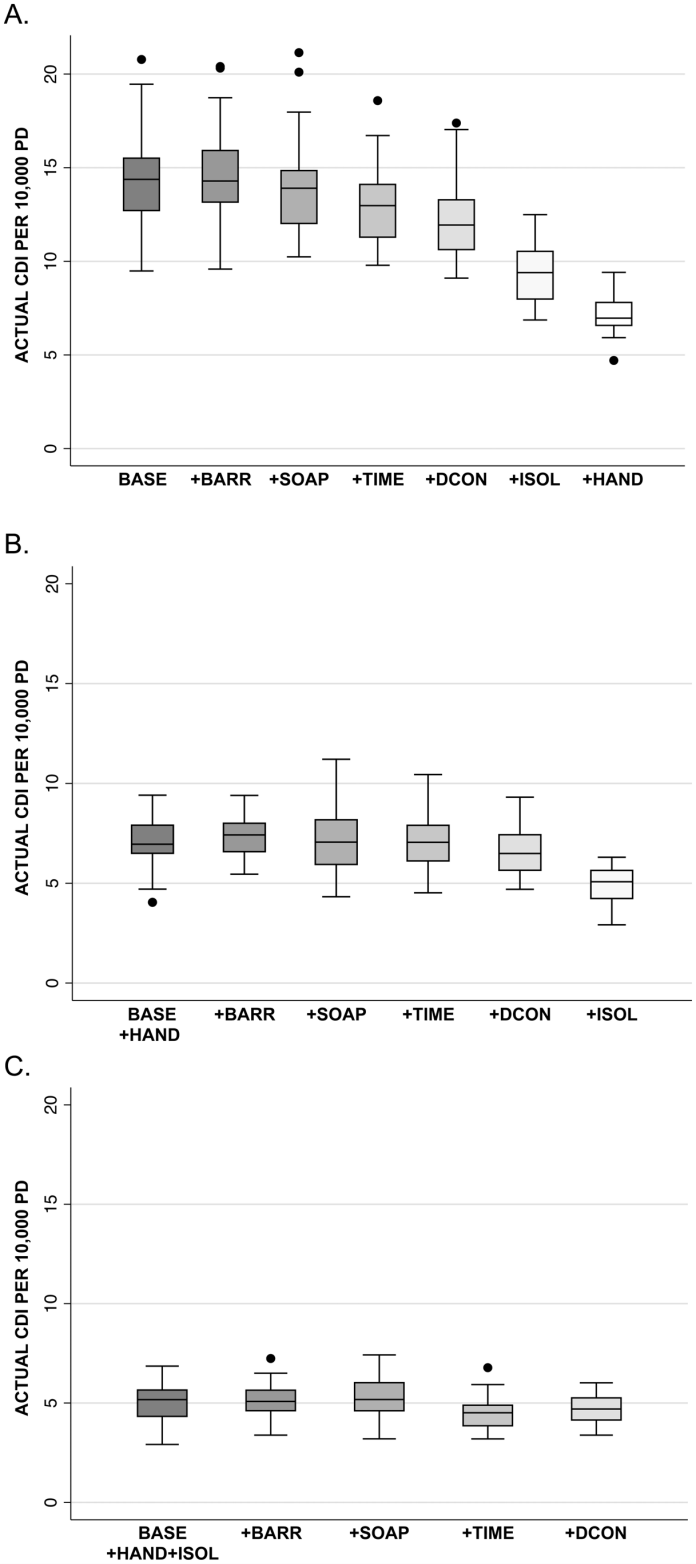
The impact of stepwise addition of bundle components on simulation results. Box plots showing the stepwise addition of bundle components from Base-Case levels to Intervention levels, one component at a time. Rates shown are actual CDI per 10,000 patient-days. The horizontal line within each box represents the median. The top and bottom of each box represent the 25th and 75th percentiles, respectively, and the bars represent the highest and lowest values within 1.5 times the interquartile range. The circles denote outliers. *Abbreviations: BASE, Base-Case scenario; BARR, improved adherence with contact/barrier precautions; SOAP, improved use of soap and water for hand hygiene for contacts with CDI patients; TIME, aggressive/early testing for C. difficile; DCON, improved environmental decontamination methods; ISOL, empiric isolation and treatment of suspected cases of CDI; HAND, improved adherence with hand hygiene.*

The results of the stepwise approach to assessing the effect of single bundle components are shown in Figure 3. The impact of each single component at INT level on the actual CDI rate was greatest for hand hygiene and empiric isolation and testing (Figure 3A); similar results were seen with the reported CDI rate (data not shown). Additional testing of each single component at INT level when combined with hand hygiene at INT level revealed a small impact only from empiric isolation and testing (Figure 3B). Increasing any other single component values to INT level (Figure 3C) or any single component to OPT level (data not shown) did not further decrease actual or reported CDI rates. No other combination of two or more bundle components demonstrated notable improvement in actual CDI rates beyond that seen with either hand hygiene alone or the combination of hand hygiene and empiric isolation and testing (data not shown).

### Sensitivity analysis

Implementation of INT and OPT levels of the complete bundle reduced actual CDI rates and *C. difficile* acquisition rates across a range of epidemiologic conditions representing *C. difficile* importation and transmission (Table 2). For reported CDI rates, the effect of INT and OPT levels of the complete bundle at low and high levels of importation and transmission mirrored those for the BASE levels; the INT scenario reduced reported CDI rates but the OPT scenario had no additional impact.

**Table 2:** Results of the simulation across the three bundled intervention scenarios while under a range of epidemiologic conditions that specify the importation and transmission rates for *C. difficile*.

	Epidemiologic	Base-Case	Typical	Optimal
	Conditions*	(No Intervention)	Intervention	Intervention
Actual CDI Rate	LOW	3.4	0.9 (74)	0.6 (82)
	BASE	14.3	3.9 (73)	2.5 (83)
	HIGH	41.9	13.6 (68)	8.4 (80)
Reported CDI Rate	LOW	2.1	0.9 (57)	0.9 (57)
	BASE	8.3	3.9 (53)	3.3 (60)
	HIGH	22.0	10.2 (54)	8.2 (63)
Acquisition Rate	LOW	37.5	12.4 (67)	8.8 (77)
	BASE	152.6	51.9 (66)	34.9 (77)
	HIGH	430.4	168.3 (61)	109.9 (74)

Rates shown are mean number of CDI cases or mean number of *C. difficile* acquistions per 10,000 patient-days. Numbers in parentheses are the percent reduction relative to the base-case scenario.

* For the epidemiologic conditions, LOW indicates low levels of *C. difficile* importation and transmission, BASE indicates base-case levels, and HIGH indicates high levels (see Table 1).

**Figure 4:**
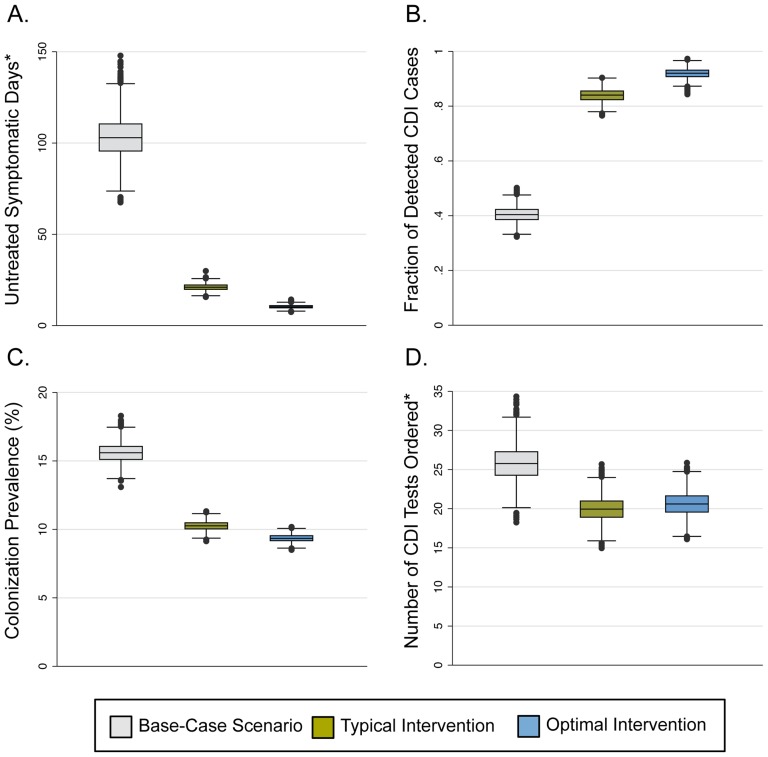
Secondary outcomes of the simulation for the three bundled intervention scenarios. Box plots showing the secondary outcomes of the simulation for each of the three bundled intervention scenarios. Grey boxes represent the Base-Case scenario (BASE), green boxes represent the Typical Intervention scenario (INT), and the blue boxes represent the Optimal Intervention scenario (OPT). Models were run using base-case values for environmental conditions. The horizontal line within each box represents the median. The top and bottom of each box represent the 25th and 75th percentiles, respectively, and the bars represent the highest and lowest values within 1.5 times the interquartile range. The circles denote outliers. *Values shown are rates per 10,000 patient-days.

Similar effects of the bundle were seen when assessing secondary outcomes. The implementation of the INT scenario resulted in large decreases in the number of untreated symptomatic days (Figure 4A), the *C. difficile* colonization prevalence (Figure 4C), and the number of CDI tests ordered (Figure 4D), along with a large increase in the fraction of CDI cases detected (Figure 4B). As with the other outcomes, increasing the intervention to OPT levels had only a small additional impact on these secondary outcomes, aside from the number of CDI tests ordered, for which there was no impact (Figure 4D).

## Discussion

We observed that "bundled" CDI control interventions dramatically reduced both *C. difficile* acquisition and CDI rates (actual and reported) in non-outbreak settings. Highly optimized bundle implementations, however, did not see proportional benefits over standard implementations due to non-linear effects and interactions between bundle components. When we examined the influence of each component individually, hand hygiene substantially affected *C. difficile* acquisition and infection rates under a wide range of conditions. Besides hand hygiene, empiric isolation and treatment of suspected CDI cases was the next most effective component; none of the other bundle interventions had a noticeable impact on acquisition or CDI rates when implemented singly, and there was little added benefit from these other interventions if enhanced hand-hygiene and empiric isolation and testing were already in place. Even when hand hygiene was modeled at lower compliance levels, it improved effectiveness regardless of which other interventions were already in place (data not shown). We hypothesize that this was due to hand hygiene being a horizontal (non-CDI specific, or universal) intervention. In contrast, *C. difficile*-targeted measures, even when elevated to extreme levels of effectiveness, showed relatively low incremental benefit when combined with other interventions. This was likely due to interactions between interventions: significant delays in test-ordering and receipt of results as well as poor sensitivity of the diagnostic test would allow for *C. difficile* “escape,” rendering intervention measures targeted to CDI less effective when implemented singly.

Most clinical studies evaluating interventions to reduce transmission of HAIs are typically single-center, before-after studies that involve a bundle of individual strategies, thereby precluding the determination of each bundle component's individual contribution and its potential for synergy, additivity, or antagonism with other components. For example, Abbett et al. report a 40% decrease in CDI incidence through the implementation of both a prevention bundle (with more than 10 components) and a treatment bundle (with more than 8 components).[23] Given the observational nature of the study and the lack of data measuring adherence to the bundle components, little can be concluded about which bundle components contributed most—if at all—to the decline in CDI that was observed.

Simulation models present one opportunity for researchers to address gaps in the empiric literature that are difficult or unethical to test in the real world. Models that allow for emergent phenomena in complex systems can evaluate bundle strategies individually and in combination to determine the presence of additive or synergistic effects. Such models are thus of practical value to hospital epidemiologists and administrators, who must often restrict resources to only one or few interventions. In addition, models provide a controlled environment in which background conditions can be precisely defined and manipulated to allow thorough evaluation of the strategy of interest.

Most published HAI models currently explore aspects of methicillin-resistant *Staphylococcus aureus* (MRSA) transmission dynamics, and few of these are constructed for the purpose of implementing one or more specific hospital interventions to reduce nosocomial transmission. Through their compartmental model of MRSA transmission, McBryde et al. [24] explored a number of different interventions in an ICU setting and found hand hygiene to be the single most effective individual intervention. Although this echoes our findings on *C. difficile* transmission, the study focused on different types of interventions (such as patient cohorting) and did not specifically explore bundled combinations of interventions. Beggs et al. [25] also studied the dynamics of hand hygiene and MRSA transmission with their stochastic Monte Carlo model and concluded, in line with our findings, that this intervention is subject to the law of diminishing returns: the greatest benefits derived from hand hygiene occur as a result of an initial, modest increase in compliance, with little added benefit from achieving very high levels of compliance. Because their model does not include, among other things, allowances for the use of gowns and gloves or for the presence of environmental reservoirs, it may not present as complete a picture as presented here, and may not generalize well to the problem of *C. difficile*. Finally, a deterministic differential equation model by D'Agata et al. [26] explored the impact of various interventions, including hand hygiene, contact precautions, a reduction in antimicrobial exposure, and screening surveillance cultures on colonization and infection prevalence for several multidrug-resistant organisms (MDRO) simultaneously, concluding that most strategies had a substantial effect on MDRO prevalence over time. Importantly, because the above models are not agent-based, they are less able to address the heterogeneity of patient-HCW contact networks or the stochasticity of interactions within the networks. The model presented here is able to produce complex, system-level dynamics and behavior patterns, which potentially can better reflect the transmission dynamics of *C. difficile* in the real world.

All models are limited by simplification and by the quality of their input parameters. In particular, our model does not address the complexity of bacterial ecosystems, the heterogeneity of innate virulence, or outbreak settings, nor does it currently evaluate antimicrobial stewardship as an intervention strategy. The latter, in particular, is an important strategy that could play a large role in the control of *C. difficile* transmission; the modeling of stewardship as an intervention is a complex process, however, and is one that we are actively pursuing as part of our future work. We also made simplifying assumptions about several processes that could impact our estimates of organism transmission. For instance, our model does not take into account CDI in HCW themselves, nor does the model allow for HCW to contaminate room environments with their hands, both of which may lead to underestimates of transmission. Our model also assumes a CDI treatment efficacy of 100%, and does not allow for recurrent CDI in patients; that said, even post-treatment, *C. difficile* organisms are still being shed into the environment, as noted in the literature.[27] This, combined with relatively short inpatient lengths of stay, likely result in little if any underestimation of in-hospital transmission. Finally, our simulation model is not intended to replace evidence produced by real-world clinical studies. We attempted here to apply agent-based simulation modeling, using the most current science and available data, to explore a range of scenarios and infection control interventions and to generate subsequent hypotheses which should provide clues to researchers faced with seemingly unlimited intervention strategies but limited resources to implement and test them.

In conclusion, our simulation model suggests that a standard CDI bundled intervention provides a marked reduction in *C. difficile* acquisitions and infections, although a highly optimized intervention provides only minimal additional benefit. Furthermore, the reported CDI rate experiences proportionately less of an impact than the actual CDI rate, which may in turn mask the true achievements of a bundled intervention. Our findings also suggest that a strong hand hygiene program and a policy of early isolation and treatment of suspected CDI cases are likely the most important components of a *C. difficile*-targeted intervention. This work should help to direct future real-world efforts to implement bundled interventions aimed at controlling CDI, minimizing costs, and identifying areas for more rigorous clinical investigation.

## Supporting Information

Appendix S1 **Detailed description of the agent-based simulation model.**
(DOCX)
