# Supplementary material for: A Simulation-Based Assessment of Strategies to Control Clostridium Difficile Transmission and Infection
Source: PLoS One. 2013 Nov 21;8(11):e80671. doi: 10.1371/journal.pone.0080671 (PMC3836736; doi:10.1371/journal.pone.0080671)
Supplement: Appendix S1 — Detailed description of the agent-based simulation model. (DOCX) [file pone.0080671.s001.docx]

**APPENDIX S1**

**A. Overview**

The simulation consists of *agent* classes to represent patients, nurses, and physicians, as well as *environment* classes to represent hospital rooms and wards. These classes operate within the context of a series of interconnected *submodels*, each of which governs a particular set of processes in the overall system and describes specific functionalities (*e.g.*, how contact isolation is implemented, or how room cleaning is performed), and the manner in which the different model components interact. The submodels are governed by rules based on our current clinical understanding of the processes involved, and are expressed mathematically to provide an explicit and technical formulation for implementation in the simulation. All of the submodels communicate and interact to produce the action, events, and output of the simulation. The following is an in-depth description of the main submodels and their key characteristics and behaviors, in an order that corresponds with Figure 1 from the manuscript.

**B. Patient Flow**


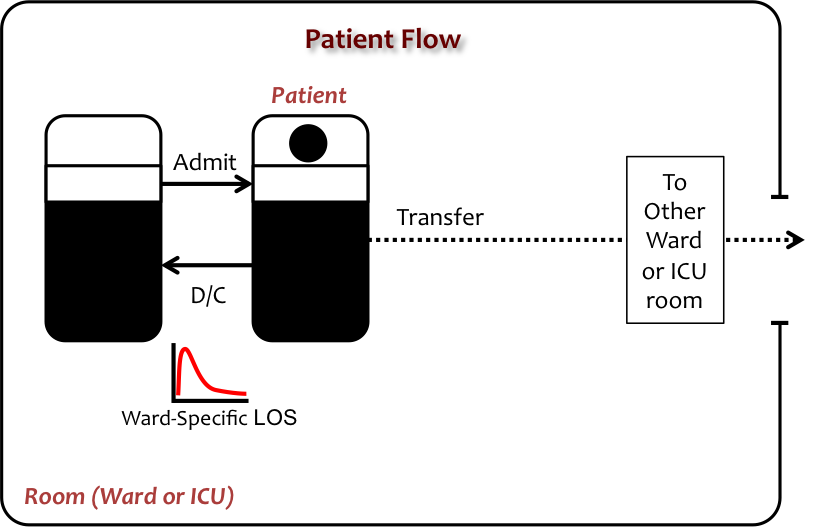
The Patient Flow submodel governed length of stay (LOS), patient flow throughout the hospital, percentage occupancy in the hospital and the degree to which length of stay was altered by *C. difficile* infection. By doing so, it also affected the degree to which infected patients contaminated the environment and thereby subjected uninfected patients to potential exposure. This impacted key factors such as the degree of patient mixing, the duration and reach of organism shedding within the hospital, and, ultimately, the reproductive number.

Admissions occurred at randomly distributed "admission times." Each day's admission times were assigned from an exponential distribution having a mean of 4 admissions per day. At each admission time, a random number of patients could be admitted from a Poisson distribution, with an average of 2 patients. If the hospital/ward was at capacity at the time of admission, then no admissions occurred. LOS was modeled based on data obtained from VA hospital ADT tables, by (a) replicating the observed distribution of LOS; (b) replicating the typical number and rate of transfers between and within regular and ICU wards; and (c) extending a simulated patient's LOS if they developed CDI during their hospitalization. Even though the extra LOS attributable to CDI may be long and may have skewed the LOS estimates of our control population, because CDI is a rare event it likely did not contribute significantly to biases in overall population parameters.

We replicated the observed distribution of LOS data by simulating a patient's LOS at the time of admission, based on the admitting ward (regular vs. ICU), from a gamma distribution. The parameters used in simulating the LOS were obtained by fitting VA LOS data to a gamma distribution. Transfer events occurred at a rate that depended on the current ward type of the patient (regular vs. ICU), with the transfers occurring either from an ICU to a ward or a ward to an ICU. The rates of transfers were parameterized so that patients were more likely to go from an ICU to a ward then from a ward to an ICU.

Hospital stays complicated by CDI were extended by adding a random amount of time to the patient’s original LOS. The additional time was sampled from a gamma distribution, having a mean of 6 days.

**C. Disease Stage / Environmental Contamination / Antibiotic Effects**


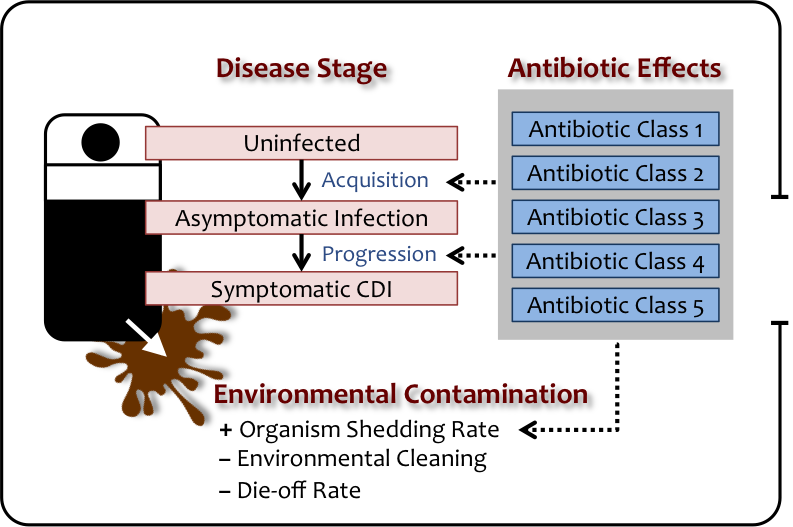
**Disease Stage.** The Disease Stage submodel describes how patients progressed through the various states of CDI, including treatment and post-treatment states. Much of the progression through the states is governed through interactions with other submodels, such as the Response/Interventions submodel. Additionally, the Disease Stage submodel specifies the impact of disease progression on other model processes, such as organism shedding rates.

Our model of CDI was based upon a breakdown of the traditional clinical view of the progression of the disease:

Uninfected → Infected/ → Infected/ → Infected/ → Infected/ → Resolved

Asympt Sympt Sympt/ Asympt/

On Treat Post-Treat

**Abbreviations: Asympt = asymptomatic; Sympt = symptomatic; Treat = treatment*

This essentially combined three independent statecharts: Infected, Symptomatic, and Treated. The separate state charts would be:

Uninfected ←→ Infected

Asymptomatic ←→ Symptomatic

No treatment → On treatment → Post-treatment

By separating the Infected statechart from the Symptomatic statechart, we allowed for the development of symptoms unrelated to *C. difficile* infection, which could have impacted resource utilization (testing, antibiotics, isolation, cohorting, etc).

The Disease Stage submodel continuously monitored the Patient class and reacted to changes in each patient's infected, symptomatic, and antibiotic treatment states. Upon receiving notification that a change in the patient state occurred, it calculated probabilities of disease change and created dynamic events to effect this change at the expiration of the event timers. The general logic flow was as follows:

Upon notification that a patient became infected, the submodel evaluated the strain to determine if it was toxigenic and, if so, computed the probability that the patient would eventually progress to a symptomatic state. The probability of progression depended on various factors including current and recent use of antibiotics. There was a positive proportion, *p^*^*, of patients who acquired C. *difficile* yet never progressed to a symptomatic state. Eligibility for progression to symptoms was determined by the probability *P^i^* for patient *i* which was calculated using the formula

$$P^{i}=\frac{1}{1+\exp\left\{-\left(\kappa_{rec}(abx_{i}^{r})\mathbf{1}_{rec}(i)+\kappa_{cur}(abx_{i}^{c})\mathbf{1}_{cur}(i)\right)\right\}}.$$

^^and ^^ are functions giving weight to antibiotics ^^ and ^^, the functions ^^ and ^^returning the current and most recent antibiotic type for patient *i*. The function ^^ is an indicator that the patient has completed at least 1 course of antibiotics while ^^ is an indicator that the patient is currently on antibiotics. Thus, with probability *P^i^*, patient *i* progressed to the symptomatic state and the submodel created a dynamic event timer that indicated the time at which the patient would transition to the symptomatic state. Symptomatic patients remained symptomatic until they were identified and treated via the Treatment submodel.

**Environmental Contamination.** C*. difficile* spores persist in the environment and can be a significant source of contamination. A dose-response model was implemented that related the number of organisms on a proportion of surfaces contaminated to the likelihood of pick-up by a health care worker (HCW). Infected patients always produced spores that were then shed into the environment, but symptomatic patients produced more; for simplicity, contaminated HCW never became infected and shed organisms into the environment themselves. The rate of shedding influenced the overall contamination of the room and the probability that a significant number of spores would eventually be brought to the room of another patient. Patients shed spores into the environment at different rates depending on their disease status and whether they were on particular antibiotics.

The following two variables quantified the contamination of each room over time:

- *E(t)* was the number of live pathogens that had settled on accessible surfaces somewhere in the room at time *t*.
- *K(t)* was the fraction of accessible surface area in the room that was contaminated at
  time *t*.

The following two simplifying assumptions were made with regard to the variables *E* and *K*: First, that the pathogen load *E* was spread evenly over the contaminated surface area; and second, that *E* was directly proportional to *K*, as defined by a proportionality constant defined below.

When an infectious patient was in a room and no decontamination events or surface touches by other individuals occurred, we assumed that the contamination variables *E* and *K* continuously changed through deposit of pathogens by the patient and natural die off of pathogens on surfaces. We assumed that removal of pathogens from surfaces by the patient (through touching) was insignificant.

*Parameters.* The following were derived through calibration to observables based on literature and expert opinion:

- *α* was the number of pathogens (spores) per unit time deposited onto accessible room surfaces by each infectious patient.
- *μ* was the rate constant for natural death of pathogens on surfaces in the room.
- *K^*^* was the equilibrium fraction of room surfaces that are contaminated when an infectious patient is in a room.

##### *Equations.* *E* changes according to the differential equation

*$\frac{dE}{dt}=\alpha-\mu E$*

which has the explicit solution

*$E(t)=E_{0}e^{-\mu t}+\frac{\alpha}{\mu}(1-e^{-\mu t})$*

In practice, *E_0_* is the value of *E* the last time it was updated for the room, and *t* is the length of time that has passed since the last update. Note that for *t → ∞*, *E* approaches an equilibrium constant, ^^. We assume that when *E* is at equilibrium, *K* is also at the equilibrium *K^*^*. This assumption, along with the assumption that *E* and *K* are directly proportional, leads to the relationship

**

So *K(t)* can be calculated directly using

**

These formulas only apply when one infectious patient is in the room, and between other external events that may change the *E* and *K* values. When an infectious patient is no longer in the room (leaves the room or ceases to be infectious), the room contamination simply decays according to

$E(t)=E_{0}e^{-\mu t}$ and $K(t)=K_{0}e^{-\mu t}$

**Environmental Decontamination.** Environmental decontamination represented cleaning of patient rooms and was implemented for two primary reasons: first, to allow for the control of transmission through the systematic removal of environmental *C. difficile* contamination; and second, to allow for the implementation of alternative methods of decontamination as one form of an infection control intervention.

Two types of decontamination were represented: (1) the cleaning that occurs on a regularly scheduled basis (*i.e.*, daily cleaning); and (2) that which occurs on discharge (*i.e.*, terminal cleaning). The effect of decontamination was to reduce the total bacterial count (*E*) and proportion of contaminated surfaces (*K*) in the room by the percent amount (*m_1_*) specified by the cleaning modality used. We defined three cleaning modalities, each having its own properties: routine regular, routine terminal, and deep terminal. The latter was reserved for the terminal cleaning of the rooms of patients identified as having *C. difficile*.

Regular cleaning events were triggered in all occupied rooms at a specified rate per day; terminal cleaning events were triggered upon patient discharge.

**Antibiotic Effects.** In this model, exposure to antibiotics affected the rate of acquisition of *C. difficile* (*i.e.*, susceptibility), the rate of progression to symptomatic disease, and the infectiousness of the patient (*i.e.*, shedding or deposition). Antibiotic effects arise from the combination of individual effects on anaerobic gastrointestinal flora and *C. difficile* and are enumerated as follows:

- Altered susceptibility to acquisition while on antibiotics
- Altered susceptibility to acquisition following antibiotic treatment
- Altered progression to symptomatic disease while on antibiotics
- Altered transmissibility through change in the organism deposit rate

We grouped antibiotics into five distinct classes based on the ability to kill *C. difficile* (for susceptible strains) and the relative magnitude of impact (expressed as odds ratios) on the four effects outlined above. One class represented antibiotics to which all *C. difficile* are presumed to be susceptible (*e.g.*, penicillins), while one class represented anti­biotics to which all *C. difficile* are considered to be resistant (*e.g.*, cephalosporins); the other three classes were created to provide variability in observed effects. Candidate estimates for the attributes of each antibiotic class were first established; we then convened an expert panel to solicit their estimates. Following a period of facilitated debate, the experts were allowed to revise their estimates based on the evidence presented. Median values of the revised estimates were used to inform the model.

*Strains and strain susceptibilities.* We created individual strains of *C. difficile* based on the set of susceptibilities to the five different classes of antibiotics. Our model was one of complete competitive strain exclusion, such that a patient/HCW could never be colonized with more than one strain. The assignment of strain was random and applied at the time of model initialization or at the time of a new importation (hospital admission of an already-infected patient).

*Antibiotic administration.* On each hospital day, there was a conditional probability of starting antibiotics (if not on them) depending on the day of hospitalization. If an antibiotic was started then the antibiotic class was selected from distributions that reflected our analysis of inpatient VA antibiotic prescribing. Antibiotic discontinuation was modeled as a random process with the time to stop drawn from an exponential distribution, with a cap at 4 weeks. There were no antibiotic combinations or changing of antibiotic regimens.

*Equations*

*Altered susceptibility to acquisition.* The main effect of antibiotics on acquisition was due to their influence on susceptibility. On contact with a contaminated HCW, the patient became infected with probability *p_A_*. The expression for *p_A_*, for patient *i*, is time-dependent and given by

$$p_{A}=\frac{1}{1+\exp\{-(b+d(\tau_{i})+\kappa_{res}(abx_{i}^{c})1_{res}(abx_{i}^{c})+\kappa_{sus}(abx_{i}^{c})1_{sus}(abx_{i}^{c})+s+c_{l}l)\}}$$

*b* represents a baseline parameter, *τ_i_* is the time (in days) since the most recent antibiotics for patient *i* and is taken to be 0 if currently on antibiotics. *d* is a function that diminishes the effects of recent antibiotics and is such that *d(τ_i_ )* decreases exponentially with *τ_i_*, but is set to 0 for *τ_i_* ≥ 28.[[1](#_ENREF_1)] *Res* and *Sus* are the classes of antibiotics to which the exposed strain is either resistant or susceptible. The function ^^, which depends on time, gives the antibiotic class at time *t*. So the functions

$$1_{res}(abx_{i}^{c}), 1_{sus}(abx_{i}^{c})$$

are indicators that ^^ is in class *Res* or *Sus,* respectively. The coefficients ^^(^^) and ^^(^^) return the odds ratio for the probability of becoming infected while on antibiotics and when the exposed strain is resistant or susceptible to ^^. *s* is a generic parameter representing an odds ratio, and is used to compare the impact of specific patient/treatment characteristics on the dynamics of the spread of *C. difficile*. *l* measures the bacterial load associated with the HCW and *c_l_* is a tuning parameter related to the bacterial load and potentially the strain.

In addition to contacts with HCW, contact with contaminated rooms can contribute to an infection. The effect of antibiotics on acquisition from a contaminated room is through the effects on *π*, the per pathogen probability of infection. The exact expression of this relationship is given by

$$\pi=\pi_{0}\exp\{d(\tau_{i})+\kappa_{res}(abx_{i}^{c})1_{res}(abx_{i}^{c})+\kappa_{sus}(abx_{i}^{c})1_{sus}(abx_{i}^{c})+s\},$$

where *π_0_* is a parameter representing a baseline probability.

*Altered progression to symptomatic disease.* Changes to antibiotic exposure altered the progression to symptomatic disease through a change in the calculated time to disease progression. If patient *i* had a time for progression to symptoms, ^^, and a change in the antibiotic status occurred at time *t_c_*, then the time remaining, ^^ - *t_c_*, was recalculated. The new time was calculated starting from *t_c_* and is given by

*$T_{S}^{i}=\beta_{l,k}^{S}(T_{S}^{i}-t_{c}), l\neq k$*

where ^^ is a time-scaling factor describing the change in the time-to-symptoms after going from antibiotic class *l* to antibiotic class *k*.

*Altered transmissibility.* Antibiotic exposure impacted infectiousness by altering the spore deposit rate, *α* , in the environmental contamination submodel. It was assumed for simplicity that the spore deposit rate was only altered in patients that were both infected and symptomatic. We used a multiplicative parameter ^^, 0 ≤ *k* ≤ 5, for adjusting the shedding rate after a patient began a course of antibiotics from class *k*. By defining ^^= 1, we express the time-dependent deposit rate, *α(t)* to be

$$\alpha(t)=\beta_{k}^{a}\alpha, if abx_{i}^{c}(t)=k, 0\leq k\leq5$$

**D. Patient Contact Events / Hand Hygiene**


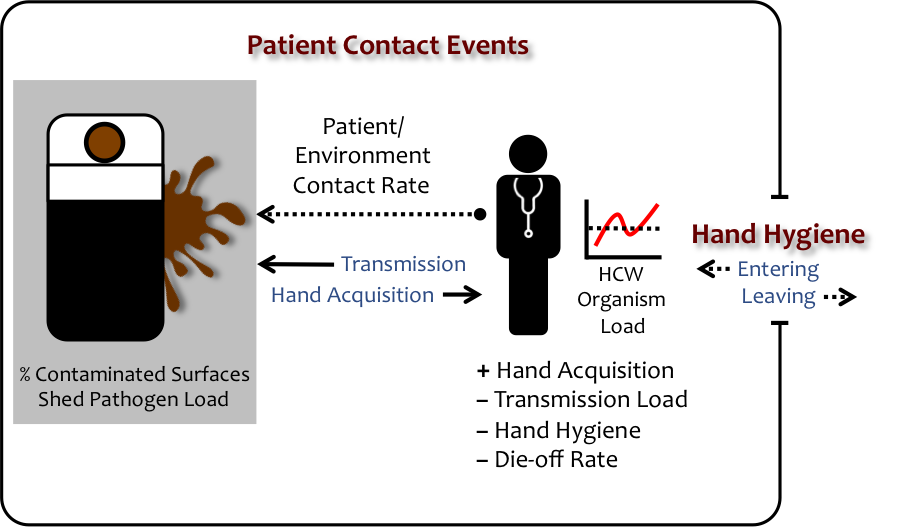
**Pick-up from environment by HCW.** For HCW, a contact event corresponded to a room visit, during which the worker may have touched potentially contaminated surfaces several times. For the purpose of the model, we included the patient as part of the "potentially contaminated room surfaces" accessible to the HCW. Our model predicted the probability that a HCW picked up contamination during a room visit as well as the level of any contamination acquired by the HCW.

*Parameters.* The following were derived through calibration to observables based on literature and expert opinion:

- *n* is the number of room surface touches by a HCW during a room visit (intensity of contact)
- *r* is the ratio of the surface area contacted per touch to the total accessible surface area of the room
- *p* is the pickup transfer efficiency (fraction of pathogens touched that successfully settled on person)

##### *Equations for calculating pick-up.* When a contact (room visit) was triggered, we calculated the current values of *E* and *K* for that room, to use in calculations. We made the following simplifying assumptions:

- Background values of *E* and *K* stayed constant during a room visit (*i.e.*, the visit was short enough that we assumed it occurred instantaneously)
- *K* was the probability that any room surface touch was on a contaminated portion of the room

Under the above assumptions, the number of surface touches that were on a contaminated surface, *a*, was distributed according to a binomial distribution, with probability parameter *K* and number parameter *n*. If *a* > 0, a transmission occurred and it followed that the probability of transmission was 1 - (1 - *K^n^*). If a transmission occurred, we calculated the total number of pathogens picked up, *b*, as

As the HCW left the room, the amount of pathogen *E* in the room was reduced by this amount (since the worker physically removed them), with a corresponding decrease in *K*. There may also have been an adjustment at this point for reducing *b* due to the use of barrier precautions (if used; see Response/Interventions, below). The value of *b* was then added to *l*, the bacterial load carried by the HCW. Hand washing upon room exit, if performed, could then reduce, *l*, as described in the next section.

*Derivation of the above equation.* Let *A_r_* be the total accessible surface area of the room, and *A_t_* be the surface area contacted by a touch, so that *r = A_t_* /*A_r_*. *E* is the total number of pathogens in the room and *KA_r_* is the area of contaminated surfaces, so *E / (KA_r_)* is the number of pathogens per area in the contaminated portion. Therefore, the number of pathogens touched is *A_t_* *E / (KA_r_)*, or *rE/K*. Multiplying by the number of contaminated touches and the pickup transfer efficiency results in the equation above. The last step results from the assumption we made that the ratio *E/K* is constant.

**Hand hygiene.** Hand hygiene behavior practiced by HCW consisted of two types: alcohol-based hand rub (ABHR) and soap and water. The effect of hand hygiene was to reduce the bacterial load on HCW hands. Although ABHR was the most commonly employed method, if *C. difficile* infection was confirmed on a patient, then the preferred method became soap and water, as ABHR lacks antimicrobial activity against *C. difficile* spores.

Hand hygiene occurred before and after patient contact, as well as at random times between patient contacts. Nurses and physicians each had their own rates of hand hygiene compliance. Additionally, when isolation precautions were in place for *C. difficile*, the HCW was more likely to engage in hand hygiene, and more likely to use soap and water.

*Parameters.* When a HCW visited a patient, hand washing occurred just before contacting the patient with probability ^^, and just after contacting the patient with probability ^^. Independent of patient contacts, hand hygiene occurred at rate ^^ throughout the shift of the HCW. The HCW in our model were limited to physicians and nurses, each having their own role-specific parameters. The corresponding parameters were denoted by ^^ and ^^ for physicians and ^^ and ^^ for nurses. Separate values for these parameters were established for both isolation rooms and non-isolation rooms, reflecting the variation in propensity to perform hand hygiene in these two room settings.

Each hand washing removed a fraction of the bacterial load from the hands of the HCW. This was implemented by multiplying the bacterial load *l* by ħ, 0<ħ<1, at the time of each hand washing. Values for ħ for both alcohol-based hand rub and soap and water were derived from the literature.[[2-4](#_ENREF_2)]

**Infection of susceptible patient from a contaminated HCW.** When a patient contact was initiated by a HCW, the HCW was first given the opportunity to perform hand hygiene. This was then followed by the possibility of becoming contaminated from the room, provided that the room was contaminated. The contact between patient and HCW was modeled by computing the probability that the HCW transmitted *C. difficile* to the patient. Transmission events occurred at the time of contact, with a probability of transmission dependent on current or recent antibiotic use of the patient, as well as the bacterial load contaminating the HCW.

**Infection of susceptible patient in contaminated room.** A susceptible patient in a room previously occupied by an infectious patient could become infected through contact with contaminated surfaces in the room. We treated this scenario differently than the HCW scenario for two reasons: first, the assumption that background values of *E* did not change during the room stay would not be accurate for extended patient stays; and second, for patients, we sought to quantify the probability of establishing infection as a result of pathogens picked up.

*Parameters.* The following were derived through calibration to observables based on literature and expert opinion:

- *v* is the patient touch rate, or the number of room surface touches by the patient per unit time
- *π* is the probability of establishing infection per pathogen picked up

*Equation for probability of infection.* Let *P(t)* be the probability of a patient becoming infected during a stay of length *t* in a contaminated room, assuming no room cleaning and no infectious person in the room during the stay. The formula is:

$$P(t)=1-\exp\left[-\frac{vpr\pi E_{0}}{\mu}(1-e^{-\mu t})\right]$$

where *E_0_* is the level of contamination of the room when the patient first enters. If the patient's length of stay in the room is known, the formula can be used to calculate the probability that infection occurs sometime during the stay. In the simulation, we generated an infection time using the distribution in the equation above, and provided that the patient remained in the room at the time of infection, they became infected at that time.

*Derivation of the above equation.* We start with the probability that an infection occurs in some small time interval Δ*t*. The number of contaminated touches in that interval is Δ*tvK*, and the number of pathogens picked up per contaminated touch is *prE/K* as derived in a previous section. Combining these, the number of pathogens picked up in the small time interval is Δ*tvprE*, and we multiply by *π* to get the probability of infection, Δ*tvprπE*. Next, it is convenient to work in terms of a survivorship-type function, *S(t)*, which is 1 - *P(t)*, or the probability that a patient is *not* infected up to time *t*. The probability of remaining uninfected up to time *t +* Δ*t* is equal to the probability of remaining uninfected up to time *t* multiplied by the probability of not being infected during the additional time Δ*t*:

$$S(t+\Delta t)=S(t)\left(1-\Delta tvpr\pi E(t)\right)$$

Using ^^ and taking the limit as Δ*t* approaches zero, we get the differential equation:

$$\frac{dS}{dt}=-vpr\pi E_{0}e^{-\mu t}S(t)$$

Solving this equation with *S(0) = 1* and converting *P(t) = 1 – S(t)* gives the equation above.

Generating an infection time followed from a general procedure for simulating random variables with a known cumulative distribution function. In this setting, for a given susceptible patient who entered a room having a pathogen load of *E_0_*, the infection time *T* (which is random) had the cumulative distribution function given by:

$$P(t)=Pr(T\leq t)=1-\exp\left[-C(1-e^{-\mu t})\right]$$

where *Pr* denotes a probability and *C = vprπE_0_/μ.*

**E. Response / Interventions**


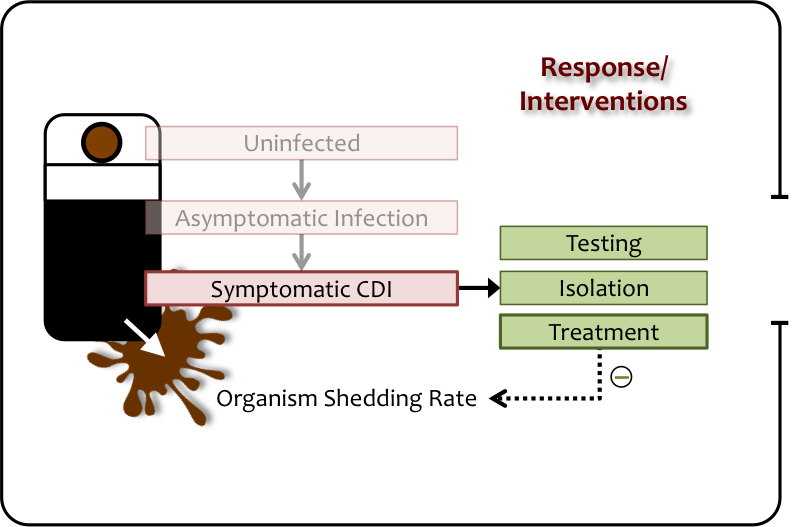
Infection control interventions are rooted in infectious disease and transmission theory. Interventions directed at *C. difficile* primarily seek to (1) interrupt the mode of transmission through contact isolation and hand hygiene, (2) destroy reservoirs through environmental cleaning and, to a certain extent, through treating patients, and (3) limit exposure time to others by implementing these interventions quickly. This submodel encompasses the implementation of the six infection control interventions and policies outlined in the manuscript, and described in detail below.

Tests for *C. difficile* infection were triggered when patients became symptomatic (*e.g.,* diarrhea). To model the presence of symptoms in the absence of *C. difficile* infection, we developed a symptomatic disease state chart with a transition rate that allowed development of diarrhea in the absence of *C. difficile* colonization.

Patients were not tested immediately after the development of symptoms, due to a delay in recognition and in ordering the test. This delay was modeled using a gamma distribution with a mean of 36 hours between the onset of symptoms and test ordering. Implementation of contact isolation was also not instantaneous. Once symptoms were recognized and a test was ordered, a time-to-isolation timer was initiated to represent the delays for sample collection and transport, processing of the test, communication of the test result, ordering of contact isolation, and implementation of contact isolation in the patient's room. This delay was also modeled using a gamma distribution with a mean of 1.75 days between the ordering of a test and the implementation of contact isolation, based on local and national VA data analysis and subject matter expert opinion. Obviously, if the *C. difficile* test result was negative, no contact isolation was implemented once the timer ended.

We modeled contact isolation based on CDC guidelines,[[5](#_ENREF_5)] which includes barrier precautions (such as glove and gown use by HCW) and single patient rooms (although all rooms in our model are single occupancy). The use of barrier precautions removed a fraction of the bacterial load picked up by the HCW during a contact event. This was implemented by multiplying the total number of pathogens picked up, *b* (see previous), by a parameter ħ, 0<ħ<1, at the time of HCW room exit. The value for ħ incorporates the effects of both gloves and gowns and was derived from the literature.[[6-8](#_ENREF_6)] The implementation of contact isolation was also associated with the enhanced use of soap and water for hand hygiene, as described later.

The *C. difficile* test sensitivity and specificity are given by σ*_sens_* and σ*_spec_*. The result of the test is random and is conditioned on the true infectious state of the patient. There are a total of four possible outcomes, as below.

If the patient has diarrhea due to *C. difficile*, the result can be a false negative (FN) or a true positive (TP):

Pr(*FN*) = 1 – σ*_sens_*, Pr(*TP*) = σ*_sens_*

If the patient does not have diarrhea due to *C. difficile*, the result can be a false positive (FP) or a true negative (TN):

Pr(*FP*) = 1 – σ*_spec_*, Pr(*TN*) = σ*_spec_*

**Aggressive/early testing.** Early testing should lead to earlier treatment, which in turn should decrease the period of symptomaticity, thereby limiting shedding and transmission of the organism.

Currently, published data are scarce on the usual time between onset of CDI symptoms and the ordering of a diagnostic test, but clinical experience and expert opinion suggested a base-case range of 12 hours to 3 days. We calibrated parameter input to reach a mean intervention value of 1 day, with an optimal value of 12 hours.

**Empiric isolation and treatment of symptomatic patients.** Contact isolation of patients and empiric treatment initiation can be implemented upon symptom recognition, rather than on the return of a positive test result, which should reduce the period of symptomatic shedding of organisms among patients with true CDI. In establishing base case values for time between onset of symptoms and treatment initiation, a few small studies with select patient populations point to a wide range (5–34 days), with a mean of roughly 5 days, when using EIA assays. We calibrated parameter input to achieve a mean intervention value of 2 days, with an optimal value of 1 day.

Published data on the usual time interval between onset of symptoms and contact isolation are scant, but a base-case value can be extrapolated from the cited interval between onset of symptoms and treatment initiation of 5 days. As for treatment initiation, we calibrated parameter input to achieve a mean intervention value of 2 days, with an optimal value of 1 day.

**Improved adherence to hand hygiene.** Hand hygiene interventions were modeled by implementing altered hand hygiene behavior with each HCW type. Changing hand hygiene behavior was accomplished by changing the values of the hand hygiene parameters ^^ and ^^ for physicians, and ^^ and ^^ for nurses, as described in the Hand Hygiene submodel, above. The values of these parameters for both isolation and non-isolation rooms were adjusted. Using literature and expert opinion, we estimated the probability of compliance with hand hygiene in the setting of contact isolation and non-isolation for both physicians and nurses in the BASE scenario, and then increased these values across the board for the INT and OPT bundle scenarios. This represented increases of 10%-20% in hand hygiene compliance for each scenario.

**Improved adherence to contact precautions.** Compliance with barrier precautions (*e.g.*, gloves and gowns) was implemented probabilistically; given an event triggering an isolation event, isolation was implemented with probability *p_I_*, with the value of *p_I_* reflecting the adherence of the staff to the isolation policy. Using literature and expert opinion, we estimated the probability of compliance with barrier precautions in the setting of contact isolation for both physicians and nurses in the BASE scenario, and increased these values for the INT and OPT bundle scenarios. The probability of compliance with barrier precautions in a non-isolation setting was considered to be considerably lower for both nurses and physicians, with no increase in either intervention scenario.

**Hand hygiene with soap & water for identified CDI cases.** The use of soap and water after contact with patients with CDI has been recommended as an important measure of *C. difficile* control. Our model implemented a hand hygiene intervention that increased the probability that a HCW used soap and water rather than an ABHR upon contact with patients identified as having CDI, and thus already in contact isolation.

**Improved environmental decontamination.** The optimal environmental disinfectant for *C. difficile* is unknown; however, hypochlorite-based disinfectants are generally considered to be the most effective. Due to their corrosive nature, their use is often restricted to areas with identified CDI patients. Quaternary ammonium compounds, the most commonly used disinfectants in hospitals, are not sporicidal. However, if implemented aggressively, they may cause a modest reduction in *C. difficile* load, if only by dilution of spores on surfaces.

As in real hospitals, environmental cleaning in our model consisted of routine daily cleaning and post-discharge terminal cleaning. Both daily and terminal cleaning in the model were performed with quaternary ammonium compounds (which provided only a small reduction in *C. difficile* load); in rooms with patients with identified CDI, however, hypochlorite was used for terminal cleaning (which achieved a substantially greater reduction in *C. difficile* load). The reduction in *C. difficile* load was reflected by the parameter *m_1_*, as described in the Environmental Decontamination section above.

To reflect a hospital-wide intervention to improve environmental decontamination, we implemented two levels of improved efficacy of cleaning (for the INT and OPT scenarios) representing increased effort of cleaning and solution exposure times. This represented increases of the value of the parameter *m_1_* by 0·05 to 0·10 for each scenario.

**Treatment of C. difficile infection.** Treatment of CDI with antibiotics served to promote the resolution of symptoms and decrease the level of organism shedding by the patient into the environment. In our model, we did not distinguish between different types of antibiotic treatment for CDI; instead, one representative treatment was modeled.

Our model of CDI progression in patients focused primarily on the transition between the defined states shown below, which governed the relative level of organism shedding by patients into the environment:


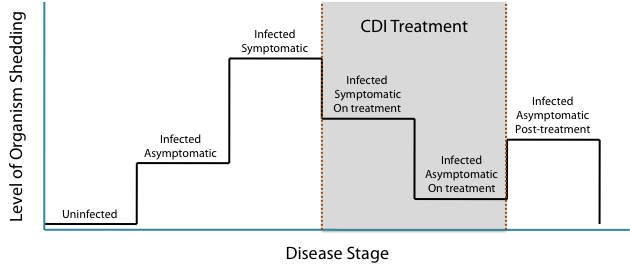
Treatment of CDI with antibiotics caused infected patients to progress from the **Infected/ Symptomatic** state to the **Infected/Symptomatic/On Treatment** state. This reduced organism shedding to a level between the Asymptomatic and Symptomatic states. At a defined point in time during CDI treatment, the patient transitioned to asymptomatic (**Infected/Asymptomatic/ On Treatment**), and organism shedding dropped to a level below the pre-treatment Asymptomatic state (but greater than 0). Once the treatment course ended, the patient transitioned to a **Post-Treatment** state where the level of shedding increased slightly, as suggested in the literature,[[9](#_ENREF_9)] and continued at this level until discharge. Actual shedding rates for each state were picked from narrow distributions with means reflecting the relative levels shown in the diagram above.

CDI treatment was started either empirically when symptoms start, or when a definitive diagnosis of CDI was made (via testing). This option was specified as part of the intervention scenario being examined (BASE, INT, or OPT). Duration of non-severe CDI treatment is typically 10 to 14 days; we simplified this by setting all treatment durations to 10 days. Although there are different treatment regimens and durations for CDI, we modeled only a single type and duration of treatment. In this simulation, we did not model resistance to CDI treatment or treatment failure.

**F. Contact Networks**


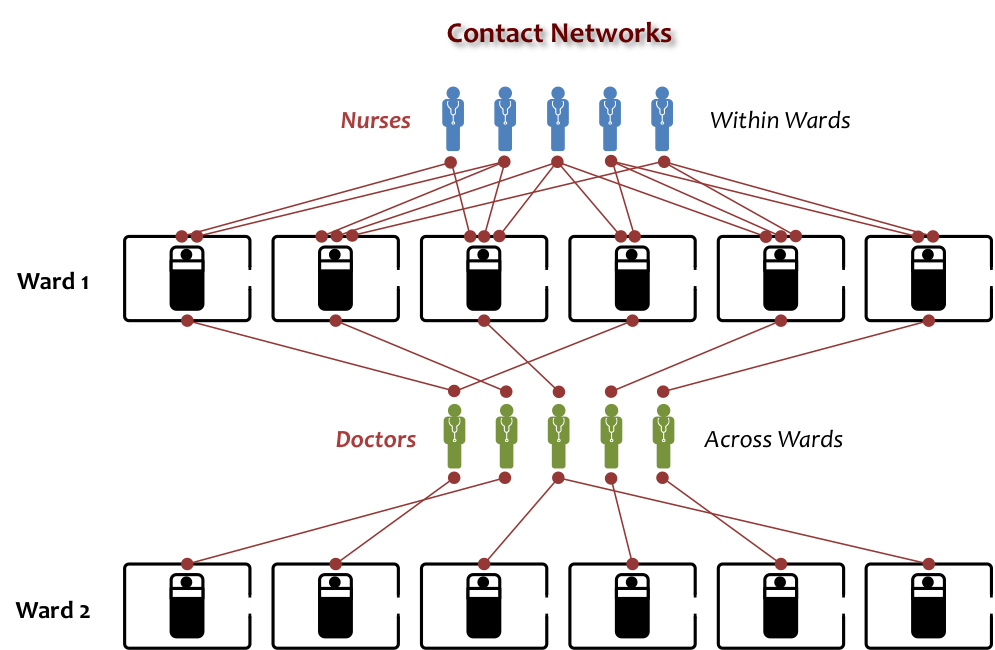
The rate of contacting a prevalent healthcare worker is a fundamental parameter in modeling transmission dynamics. The role of the HCW influences the effective prevalence to which any one individual is exposed. Simple dynamic models often assume that the probability of contact between prevalent and susceptible patients is uniform and homogeneous, an assumption that is not true in the real world. The different sizes of groups and connections between individuals in this agent-based model essentially relaxed the assumptions of more simple approaches. This allowed for the possibility of more interesting and realistic dynamics.

The contact network was the scaffolding upon which transmission occurred. It defined the connections between patients, HCWs, and rooms. It can be described mathematically as a graph with three types of nodes (vertices): *p_1_, p_2_, …, p_Np_* representing the patients; *h_1_, h_2_, …, h_Nh_* representing the HCW; and *r_1_, r_2_, …, r_Nr_* representing the rooms in the hospital. The number of patients, *N_p_*, was random and evolved over time, influenced by the process of admission and
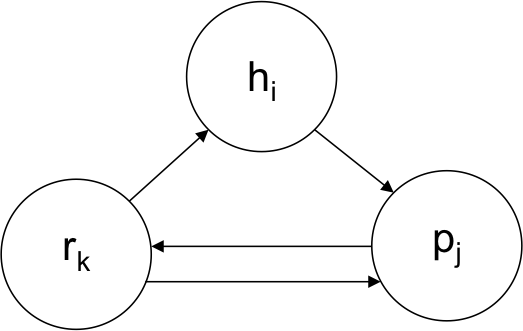
discharge. Thus, the vertices in the graph changed over time. The directions of the arcs between any pair of vertices indicate the direction of transmission of a particular pathogen, as demonstrated in the figure below:

If patient *p_j_* occupied room *r_k_* and was assigned healthcare worker *h_i_*, then it was possible for the pathogen to move from *p_j_* to *r_k_* via shedding of the pathogen into the room, or from *r_k_* to *h_i_*, a HCW picking up the pathogen from a contaminated room. The patient *p_j_* could become infected either from a contaminated HCW *h_i_*, or a contaminated room, *r_k_*. It is important to note that the arcs do not imply an actual transmission event, but the possible paths for a transmission event, given the current contact network structure. Due to patient flow governed by the Patient Flow submodel and the changes in healthcare assignments, the contact structure changed over time.

Dynamics over time are better understood by considering both types of HCW, physicians and nurses. They differed in important ways in their connections with patients. Physicians were assigned patients from multiple wards, while nurses were only assigned patients from a single ward. Additionally, the number of physicians per patient was different from the number of nurses per patient. At the time that arcs were created, each eligible patient was randomly assigned two physicians from the list of possible physicians. Each patient was also independently assigned min(N, N_tot_) nurses, where the minimum is between N_tot_, the total number of nurses available, and N. N is a random variable having the distribution

N ~ Pois(*μ_N_* – N_min_) + N_min_

Here, *μ_N_* is the average number of nurses on a ward and N_min_ is the minimum number of nurses allowed per patient. Arcs between rooms and patients changed when a patient either left or entered a room. When there was an admission or transfer, a patient was randomly assigned to an available room. If there were no rooms available, then the admission/transfer event did not take place.

**G. References (see manuscript for complete list)**

1. Hensgens MP, Goorhuis A, Dekkers OM, Kuijper EJ (2012) Time interval of increased risk for Clostridium difficile infection after exposure to antibiotics. J Antimicrob Chemother 67: 742-748.

2. Bettin K, Clabots C, Mathie P, Willard K, Gerding DN (1994) Effectiveness of liquid soap vs. chlorhexidine gluconate for the removal of Clostridium difficile from bare hands and gloved hands. Infect Control Hosp Epidemiol 15: 697-702.

3. Jabbar U, Leischner J, Kasper D, Gerber R, Sambol SP, et al. (2010) Effectiveness of alcohol-based hand rubs for removal of Clostridium difficile spores from hands. Infect Control Hosp Epidemiol 31: 565-570.

4. Oughton MT, Loo VG, Dendukuri N, Fenn S, Libman MD (2009) Hand hygiene with soap and water is superior to alcohol rub and antiseptic wipes for removal of Clostridium difficile. Infect Control Hosp Epidemiol 30: 939-944.

5. Siegel J, Rhinehart E, Jackson M, Chiarello L, and the Healthcare Infection Control Practices Advisory Committee (2007) 2007 Guideline for Isolation Precautions: Preventing Transmission of Infectious Agents in Healthcare Settings. At <http://www.cdc.gov/ncidod/dhqp/pdf/isolation2007.pdf> (last accessed 8/1/2013).

6. Hayden MK, Blom DW, Lyle EA, Moore CG, Weinstein RA (2008) Risk of hand or glove contamination after contact with patients colonized with vancomycin-resistant enterococcus or the colonized patients' environment. Infect Control Hosp Epidemiol 29: 149-154.

7. Olsen RJ, Lynch P, Coyle MB, Cummings J, Bokete T, et al. (1993) Examination gloves as barriers to hand contamination in clinical practice. JAMA 270: 350-353.

8. Tenorio AR, Badri SM, Sahgal NB, Hota B, Matushek M, et al. (2001) Effectiveness of gloves in the prevention of hand carriage of vancomycin-resistant enterococcus species by health care workers after patient care. Clin Infect Dis 32: 826-829.

9. Sethi AK, Al-Nassir WN, Nerandzic MM, Bobulsky GS, Donskey CJ (2010) Persistence of skin contamination and environmental shedding of Clostridium difficile during and after treatment of C. difficile infection. Infect Control Hosp Epidemiol 31: 21-27.
